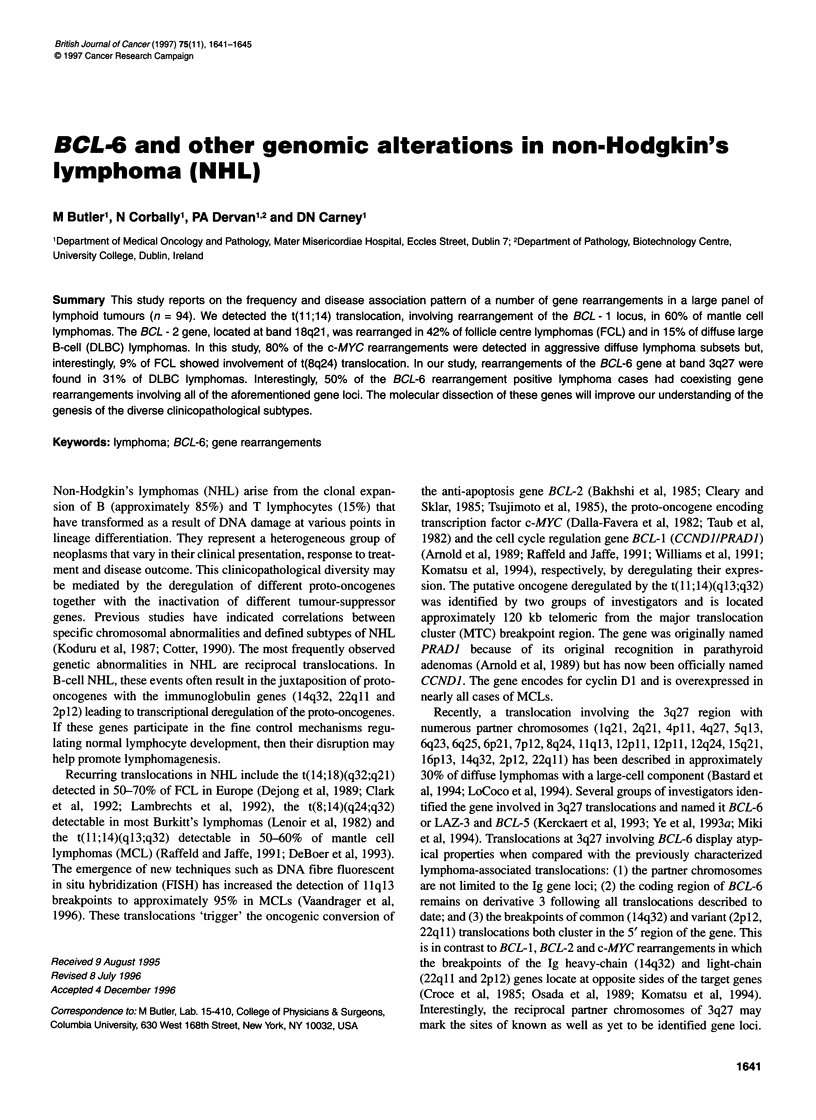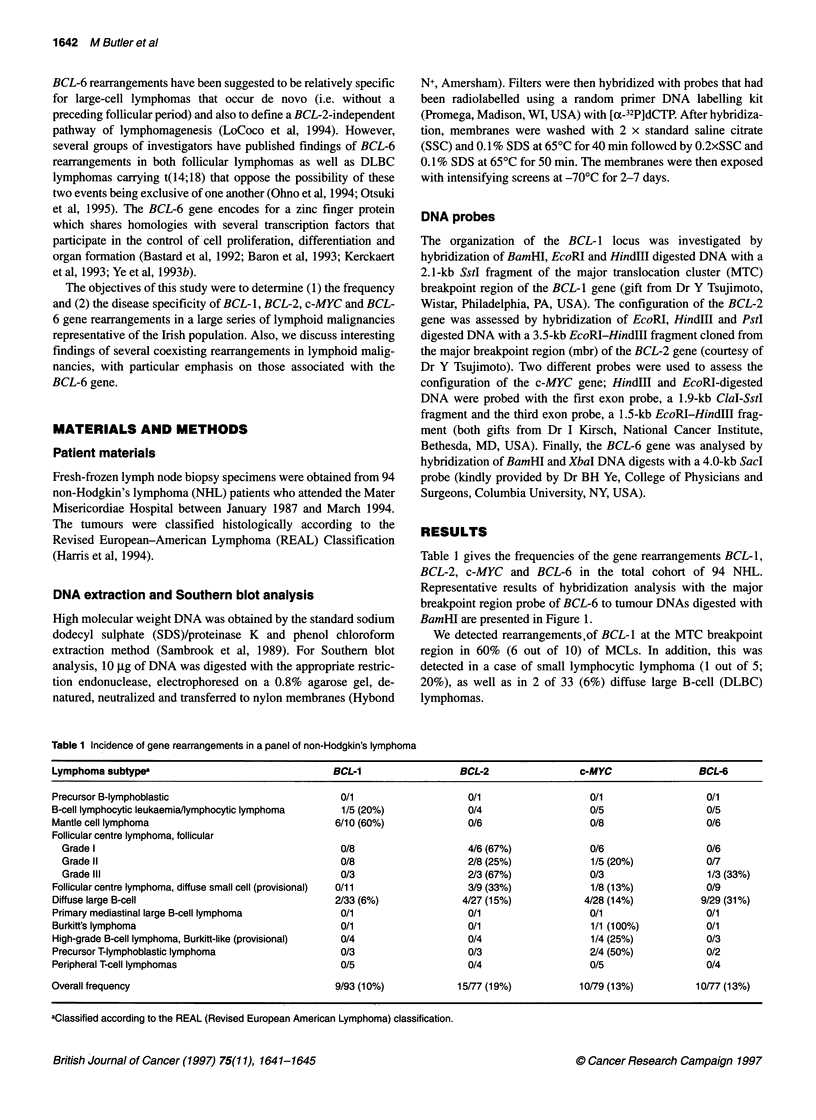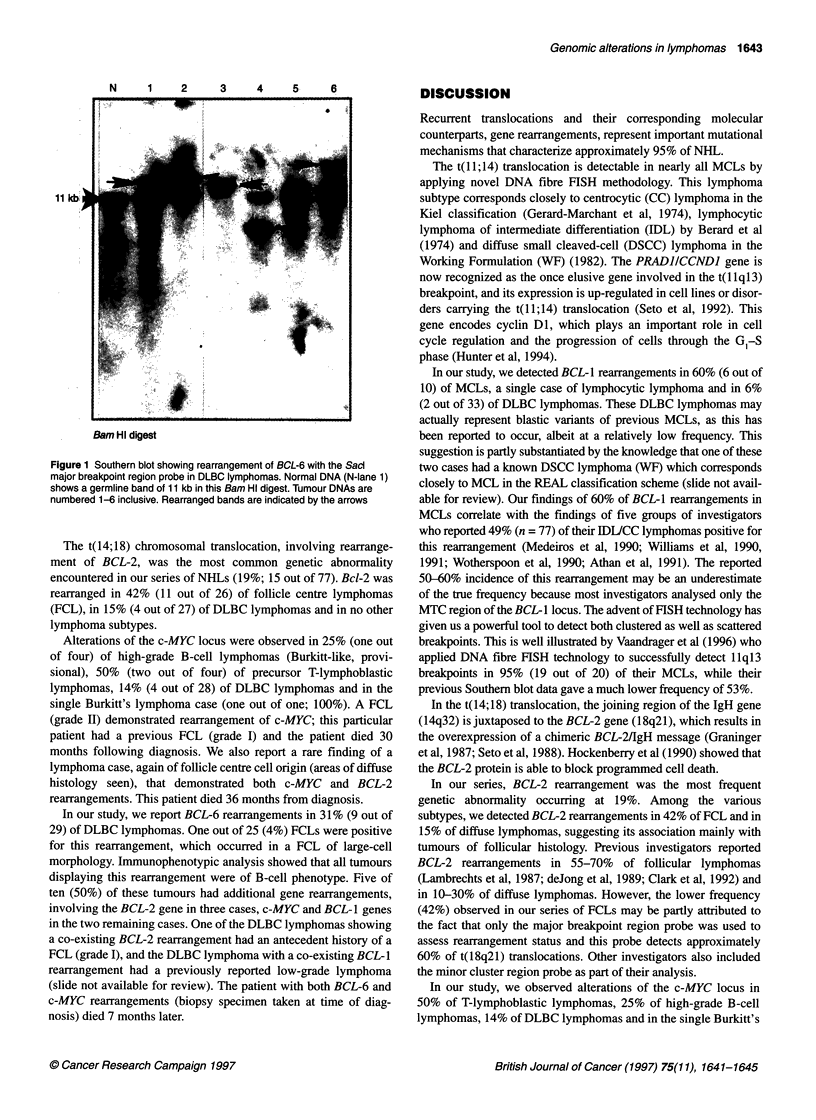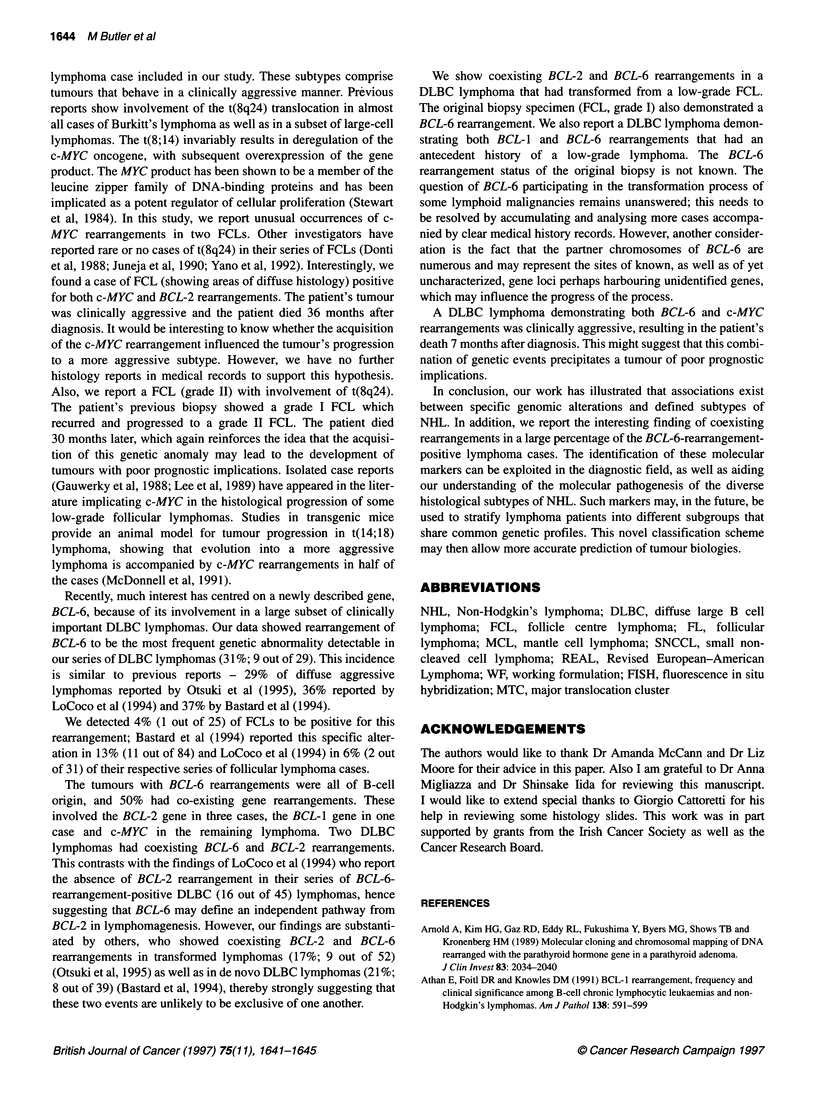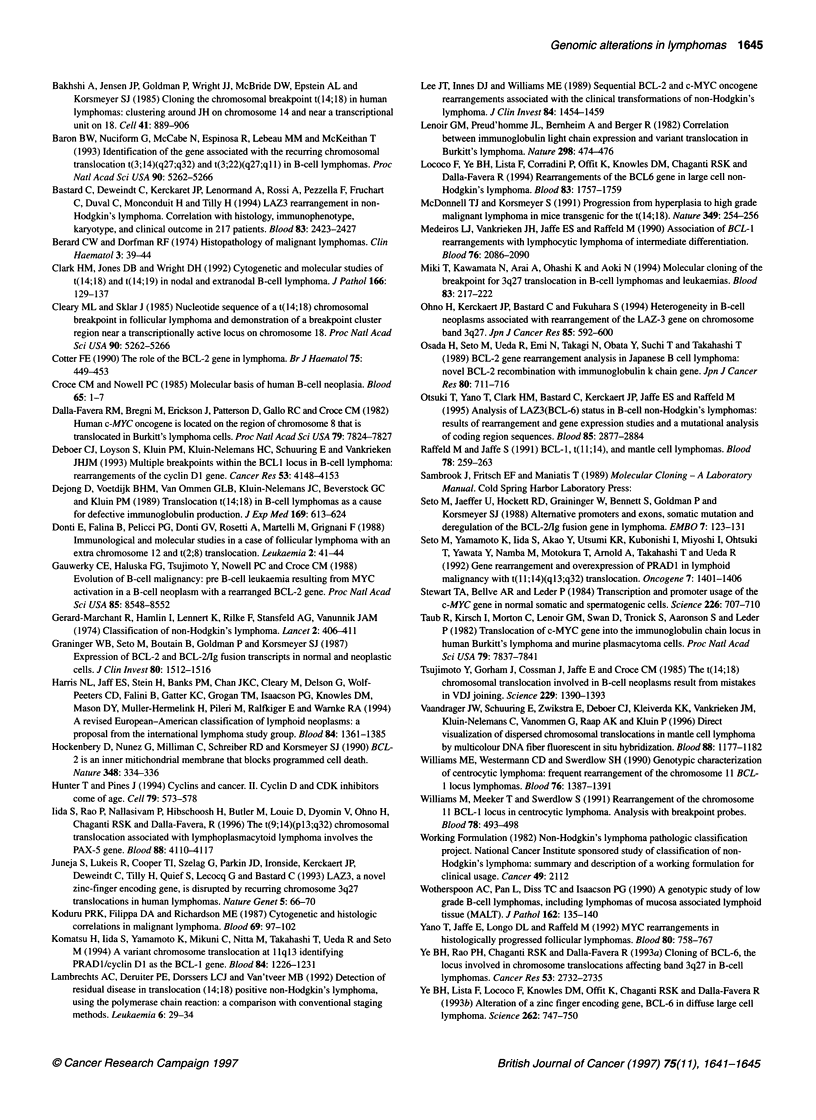# BCL-6 and other genomic alterations in non-Hodgkin's lymphoma (NHL).

**DOI:** 10.1038/bjc.1997.279

**Published:** 1997

**Authors:** M. Butler, N. Corbally, P. A. Dervan, D. N. Carney

**Affiliations:** Department of Medical Oncology and Pathology, Mater Misericordiae Hospital, Dublin, Ireland.

## Abstract

**Images:**


British Journal of Cancer (1997) 75(11), 1641–1645
© 1997 Cancer Research Campaign

### *BCL-6* and other genomic alterations in non-Hodgkin's lymphoma (NHL)

M Butler[1], N Corbally[1], PA Dervan[1,2] and DN Carney[1]

[1]Department of Medical Oncology and Pathology, Mater Misericordiae Hospital, Eccles Street, Dublin 7; [2]Department of Pathology, Biotechnology Centre, University College, Dublin, Ireland

**Summary** This study reports on the frequency and disease association pattern of a number of gene rearrangements in a large panel of lymphoid tumours ($n$ = 94). We detected the t(11;14) translocation, involving rearrangement of the *BCL* - 1 locus, in 60% of mantle cell lymphomas. The *BCL* - 2 gene, located at band 18q21, was rearranged in 42% of follicle centre lymphomas (FCL) and in 15% of diffuse large B-cell (DLBC) lymphomas. In this study, 80% of the c-*MYC* rearrangements were detected in aggressive diffuse lymphoma subsets but, interestingly, 9% of FCL showed involvement of t(8q24) translocation. In our study, rearrangements of the *BCL*-6 gene at band 3q27 were found in 31% of DLBC lymphomas. Interestingly, 50% of the *BCL*-6 rearrangement positive lymphoma cases had coexisting gene rearrangements involving all of the aforementioned gene loci. The molecular dissection of these genes will improve our understanding of the genesis of the diverse clinicopathological subtypes.

**Keywords:** lymphoma; *BCL*-6; gene rearrangements

Non-Hodgkin's lymphomas (NHL) arise from the clonal expansion of B (approximately 85%) and T lymphocytes (15%) that have transformed as a result of DNA damage at various points in lineage differentiation. They represent a heterogeneous group of neoplasms that vary in their clinical presentation, response to treatment and disease outcome. This clinicopathological diversity may be mediated by the deregulation of different proto-oncogenes together with the inactivation of different tumour-suppressor genes. Previous studies have indicated correlations between specific chromosomal abnormalities and defined subtypes of NHL (Koduru et al, 1987; Cotter, 1990). The most frequently observed genetic abnormalities in NHL are reciprocal translocations. In B-cell NHL, these events often result in the juxtaposition of proto-oncogenes with the immunoglobulin genes (14q32, 22q11 and 2p12) leading to transcriptional deregulation of the proto-oncogenes. If these genes participate in the fine control mechanisms regulating normal lymphocyte development, then their disruption may help promote lymphomagenesis.

Recurring translocations in NHL include the t(14;18)(q32;q21) detected in 50–70% of FCL in Europe (Dejong et al, 1989; Clark et al, 1992; Lambrechts et al, 1992), the t(8;14)(q24;q32) detectable in most Burkitt's lymphomas (Lenoir et al, 1982) and the t(11;14)(q13;q32) detectable in 50–60% of mantle cell lymphomas (MCL) (Raffeld and Jaffe, 1991; DeBoer et al, 1993). The emergence of new techniques such as DNA fibre fluorescent in situ hybridization (FISH) has increased the detection of 11q13 breakpoints to approximately 95% in MCLs (Vaandrager et al, 1996). These translocations 'trigger' the oncogenic conversion of the anti-apoptosis gene *BCL*-2 (Bakhshi et al, 1985; Cleary and Sklar, 1985; Tsujimoto et al, 1985), the proto-oncogene encoding transcription factor c-*MYC* (Dalla-Favera et al, 1982; Taub et al, 1982) and the cell cycle regulation gene *BCL*-1 (*CCND1*/*PRAD1*) (Arnold et al, 1989; Raffeld and Jaffe, 1991; Williams et al, 1991; Komatsu et al, 1994), respectively, by deregulating their expression. The putative oncogene deregulated by the t(11;14)(q13;q32) was identified by two groups of investigators and is located approximately 120 kb telomeric from the major translocation cluster (MTC) breakpoint region. The gene was originally named *PRAD1* because of its original recognition in parathyroid adenomas (Arnold et al, 1989) but has now been officially named *CCND1*. The gene encodes for cyclin D1 and is overexpressed in nearly all cases of MCLs.

Recently, a translocation involving the 3q27 region with numerous partner chromosomes (1q21, 2q21, 4p11, 4q27, 5q13, 6q23, 6q25, 6p21, 7p12, 8q24, 11q13, 12p11, 12p11, 12q24, 15q21, 16p13, 14q32, 2p12, 22q11) has been described in approximately 30% of diffuse lymphomas with a large-cell component (Bastard et al, 1994; LoCoco et al, 1994). Several groups of investigators identified the gene involved in 3q27 translocations and named it *BCL*-6 or LAZ-3 and *BCL*-5 (Kerckaert et al, 1993; Ye et al, 1993*a*; Miki et al, 1994). Translocations at 3q27 involving *BCL*-6 display atypical properties when compared with the previously characterized lymphoma-associated translocations: (1) the partner chromosomes are not limited to the Ig gene loci; (2) the coding region of *BCL*-6 remains on derivative 3 following all translocations described to date; and (3) the breakpoints of common (14q32) and variant (2p12, 22q11) translocations both cluster in the 5′ region of the gene. This is in contrast to *BCL*-1, *BCL*-2 and c-*MYC* rearrangements in which the breakpoints of the Ig heavy-chain (14q32) and light-chain (22q11 and 2p12) genes locate at opposite sides of the target genes (Croce et al, 1985; Osada et al, 1989; Komatsu et al, 1994). Interestingly, the reciprocal partner chromosomes of 3q27 may mark the sites of known as well as yet to be identified gene loci.

*Received 9 August 1995*
*Revised 8 July 1996*
*Accepted 4 December 1996*

*Correspondence to:* M Butler, Lab. 15-410, College of Physicians & Surgeons, Columbia University, 630 West 168th Street, New York, NY 10032, USA

*BCL*-6 rearrangements have been suggested to be relatively specific for large-cell lymphomas that occur de novo (i.e. without a preceding follicular period) and also to define a *BCL*-2-independent pathway of lymphomagenesis (LoCoco et al, 1994). However, several groups of investigators have published findings of *BCL*-6 rearrangements in both follicular lymphomas as well as DLBC lymphomas carrying t(14;18) that oppose the possibility of these two events being exclusive of one another (Ohno et al, 1994; Otsuki et al, 1995). The *BCL*-6 gene encodes for a zinc finger protein which shares homologies with several transcription factors that participate in the control of cell proliferation, differentiation and organ formation (Bastard et al, 1992; Baron et al, 1993; Kerckaert et al, 1993; Ye et al, 1993*b*).

The objectives of this study were to determine (1) the frequency and (2) the disease specificity of *BCL*-1, *BCL*-2, c-*MYC* and *BCL*-6 gene rearrangements in a large series of lymphoid malignancies representative of the Irish population. Also, we discuss interesting findings of several coexisting rearrangements in lymphoid malignancies, with particular emphasis on those associated with the *BCL*-6 gene.

### MATERIALS AND METHODS

### Patient materials

Fresh-frozen lymph node biopsy specimens were obtained from 94 non-Hodgkin's lymphoma (NHL) patients who attended the Mater Misericordiae Hospital between January 1987 and March 1994. The tumours were classified histologically according to the Revised European–American Lymphoma (REAL) Classification (Harris et al, 1994).

### DNA extraction and Southern blot analysis

High molecular weight DNA was obtained by the standard sodium dodecyl sulphate (SDS)/proteinase K and phenol chloroform extraction method (Sambrook et al, 1989). For Southern blot analysis, 10 μg of DNA was digested with the appropriate restriction endonuclease, electrophoresed on a 0.8% agarose gel, denatured, neutralized and transferred to nylon membranes (Hybond $N^+$, Amersham). Filters were then hybridized with probes that had been radiolabelled using a random primer DNA labelling kit (Promega, Madison, WI, USA) with [$\alpha$-$^{32}$P]dCTP. After hybridization, membranes were washed with 2 × standard saline citrate (SSC) and 0.1% SDS at 65°C for 40 min followed by 0.2×SSC and 0.1% SDS at 65°C for 50 min. The membranes were then exposed with intensifying screens at –70°C for 2–7 days.

### DNA probes

The organization of the *BCL*-1 locus was investigated by hybridization of *Bam*HI, *Eco*RI and *Hin*dIII digested DNA with a 2.1-kb *Sst*I fragment of the major translocation cluster (MTC) breakpoint region of the *BCL*-1 gene (gift from Dr Y Tsujimoto, Wistar, Philadelphia, PA, USA). The configuration of the *BCL*-2 gene was assessed by hybridization of *Eco*RI, *Hin*dIII and *Pst*I digested DNA with a 3.5-kb *Eco*RI–*Hin*dIII fragment cloned from the major breakpoint region (mbr) of the *BCL*-2 gene (courtesy of Dr Y Tsujimoto). Two different probes were used to assess the configuration of the c-*MYC* gene; *Hin*dIII and *Eco*RI-digested DNA were probed with the first exon probe, a 1.9-kb *Cla*I-*Sst*I fragment and the third exon probe, a 1.5-kb *Eco*RI–*Hin*dIII fragment (both gifts from Dr I Kirsch, National Cancer Institute, Bethesda, MD, USA). Finally, the *BCL*-6 gene was analysed by hybridization of *Bam*HI and *Xba*I DNA digests with a 4.0-kb *Sac*I probe (kindly provided by Dr BH Ye, College of Physicians and Surgeons, Columbia University, NY, USA).

### RESULTS

Table 1 gives the frequencies of the gene rearrangements *BCL*-1, *BCL*-2, c-*MYC* and *BCL*-6 in the total cohort of 94 NHL. Representative results of hybridization analysis with the major breakpoint region probe of *BCL*-6 to tumour DNAs digested with *Bam*HI are presented in Figure 1.

We detected rearrangements of *BCL*-1 at the MTC breakpoint region in 60% (6 out of 10) of MCLs. In addition, this was detected in a case of small lymphocytic lymphoma (1 out of 5; 20%), as well as in 2 of 33 (6%) diffuse large B-cell (DLBC) lymphomas.

**Table 1** Incidence of gene rearrangements in a panel of non-Hodgkin's lymphoma

| Lymphoma subtype[a] | *BCL*-1 | *BCL*-2 | c-*MYC* | *BCL*-6 |
|---|---|---|---|---|
| Precursor B-lymphoblastic | 0/1 | 0/1 | 0/1 | 0/1 |
| B-cell lymphocytic leukaemia/lymphocytic lymphoma | 1/5 (20%) | 0/4 | 0/5 | 0/5 |
| Mantle cell lymphoma | 6/10 (60%) | 0/6 | 0/8 | 0/6 |
| Follicular centre lymphoma, follicular | | | | |
| Grade I | 0/8 | 4/6 (67%) | 0/6 | 0/6 |
| Grade II | 0/8 | 2/8 (25%) | 1/5 (20%) | 0/7 |
| Grade III | 0/3 | 2/3 (67%) | 0/3 | 1/3 (33%) |
| Follicular centre lymphoma, diffuse small cell (provisional) | 0/11 | 3/9 (33%) | 1/8 (13%) | 0/9 |
| Diffuse large B-cell | 2/33 (6%) | 4/27 (15%) | 4/28 (14%) | 9/29 (31%) |
| Primary mediastinal large B-cell lymphoma | 0/1 | 0/1 | 0/1 | 0/1 |
| Burkitt's lymphoma | 0/1 | 0/1 | 1/1 (100%) | 0/1 |
| High-grade B-cell lymphoma, Burkitt-like (provisional) | 0/4 | 0/4 | 1/4 (25%) | 0/3 |
| Precursor T-lymphoblastic lymphoma | 0/3 | 0/3 | 2/4 (50%) | 0/2 |
| Peripheral T-cell lymphomas | 0/5 | 0/4 | 0/5 | 0/4 |
| Overall frequency | 9/93 (10%) | 15/77 (19%) | 10/79 (13%) | 10/77 (13%) |

[a]Classified according to the REAL (Revised European American Lymphoma) classification.

© Cancer Research Campaign 1997

**Figure 1** Southern blot showing rearrangement of *BCL*-6 with the *SacI* major breakpoint region probe in DLBC lymphomas. Normal DNA (N-lane 1) shows a germline band of 11 kb in this *Bam* HI digest. Tumour DNAs are numbered 1–6 inclusive. Rearranged bands are indicated by the arrows

The t(14;18) chromosomal translocation, involving rearrangement of *BCL*-2, was the most common genetic abnormality encountered in our series of NHLs (19%; 15 out of 77). *Bcl*-2 was rearranged in 42% (11 out of 26) of follicle centre lymphomas (FCL), in 15% (4 out of 27) of DLBC lymphomas and in no other lymphoma subtypes.

Alterations of the c-*MYC* locus were observed in 25% (one out of four) of high-grade B-cell lymphomas (Burkitt-like, provisional), 50% (two out of four) of precursor T-lymphoblastic lymphomas, 14% (4 out of 28) of DLBC lymphomas and in the single Burkitt's lymphoma case (one out of one; 100%). A FCL (grade II) demonstrated rearrangement of c-*MYC*; this particular patient had a previous FCL (grade I) and the patient died 30 months following diagnosis. We also report a rare finding of a lymphoma case, again of follicle centre cell origin (areas of diffuse histology seen), that demonstrated both c-*MYC* and *BCL*-2 rearrangements. This patient died 36 months from diagnosis.

In our study, we report *BCL*-6 rearrangements in 31% (9 out of 29) of DLBC lymphomas. One out of 25 (4%) FCLs were positive for this rearrangement, which occurred in a FCL of large-cell morphology. Immunophenotypic analysis showed that all tumours displaying this rearrangement were of B-cell phenotype. Five of ten (50%) of these tumours had additional gene rearrangements, involving the *BCL*-2 gene in three cases, c-*MYC* and *BCL*-1 genes in the two remaining cases. One of the DLBC lymphomas showing a co-existing *BCL*-2 rearrangement had an antecedent history of a FCL (grade I), and the DLBC lymphoma with a co-existing *BCL*-1 rearrangement had a previously reported low-grade lymphoma (slide not available for review). The patient with both *BCL*-6 and c-*MYC* rearrangements (biopsy specimen taken at time of diagnosis) died 7 months later.

### DISCUSSION

Recurrent translocations and their corresponding molecular counterparts, gene rearrangements, represent important mutational mechanisms that characterize approximately 95% of NHL.

The t(11;14) translocation is detectable in nearly all MCLs by applying novel DNA fibre FISH methodology. This lymphoma subtype corresponds closely to centrocytic (CC) lymphoma in the Kiel classification (Gerard-Marchant et al, 1974), lymphocytic lymphoma of intermediate differentiation (IDL) by Berard et al (1974) and diffuse small cleaved-cell (DSCC) lymphoma in the Working Formulation (WF) (1982). The *PRAD1/CCND1* gene is now recognized as the once elusive gene involved in the t(11q13) breakpoint, and its expression is up-regulated in cell lines or disorders carrying the t(11;14) translocation (Seto et al, 1992). This gene encodes cyclin D1, which plays an important role in cell cycle regulation and the progression of cells through the $G_1$–S phase (Hunter et al, 1994).

In our study, we detected *BCL*-1 rearrangements in 60% (6 out of 10) of MCLs, a single case of lymphocytic lymphoma and in 6% (2 out of 33) of DLBC lymphomas. These DLBC lymphomas may actually represent blastic variants of previous MCLs, as this has been reported to occur, albeit at a relatively low frequency. This suggestion is partly substantiated by the knowledge that one of these two cases had a known DSCC lymphoma (WF) which corresponds closely to MCL in the REAL classification scheme (slide not available for review). Our findings of 60% of *BCL*-1 rearrangements in MCLs correlate with the findings of five groups of investigators who reported 49% ($n$ = 77) of their IDL/CC lymphomas positive for this rearrangement (Medeiros et al, 1990; Williams et al, 1990, 1991; Wotherspoon et al, 1990; Athan et al, 1991). The reported 50–60% incidence of this rearrangement may be an underestimate of the true frequency because most investigators analysed only the MTC region of the *BCL*-1 locus. The advent of FISH technology has given us a powerful tool to detect both clustered as well as scattered breakpoints. This is well illustrated by Vaandrager et al (1996) who applied DNA fibre FISH technology to successfully detect 11q13 breakpoints in 95% (19 out of 20) of their MCLs, while their previous Southern blot data gave a much lower frequency of 53%.

In the t(14;18) translocation, the joining region of the IgH gene (14q32) is juxtaposed to the *BCL*-2 gene (18q21), which results in the overexpression of a chimeric *BCL*-2/IgH message (Graninger et al, 1987; Seto et al, 1988). Hockenberry et al (1990) showed that the *BCL*-2 protein is able to block programmed cell death.

In our series, *BCL*-2 rearrangement was the most frequent genetic abnormality occurring at 19%. Among the various subtypes, we detected *BCL*-2 rearrangements in 42% of FCL and in 15% of diffuse lymphomas, suggesting its association mainly with tumours of follicular histology. Previous investigators reported *BCL*-2 rearrangements in 55–70% of follicular lymphomas (Lambrechts et al, 1987; deJong et al, 1989; Clark et al, 1992) and in 10–30% of diffuse lymphomas. However, the lower frequency (42%) observed in our series of FCLs may be partly attributed to the fact that only the major breakpoint region probe was used to assess rearrangement status and this probe detects approximately 60% of t(18q21) translocations. Other investigators also included the minor cluster region probe as part of their analysis.

In our study, we observed alterations of the c-*MYC* locus in 50% of T-lymphoblastic lymphomas, 25% of high-grade B-cell lymphomas, 14% of DLBC lymphomas and in the single Burkitt's

© Cancer Research Campaign 1997

lymphoma case included in our study. These subtypes comprise tumours that behave in a clinically aggressive manner. Previous reports show involvement of the t(8q24) translocation in almost all cases of Burkitt's lymphoma as well as in a subset of large-cell lymphomas. The t(8;14) invariably results in deregulation of the c-*MYC* oncogene, with subsequent overexpression of the gene product. The *MYC* product has been shown to be a member of the leucine zipper family of DNA-binding proteins and has been implicated as a potent regulator of cellular proliferation (Stewart et al, 1984). In this study, we report unusual occurrences of c-*MYC* rearrangements in two FCLs. Other investigators have reported rare or no cases of t(8q24) in their series of FCLs (Donti et al, 1988; Juneja et al, 1990; Yano et al, 1992). Interestingly, we found a case of FCL (showing areas of diffuse histology) positive for both c-*MYC* and *BCL*-2 rearrangements. The patient's tumour was clinically aggressive and the patient died 36 months after diagnosis. It would be interesting to know whether the acquisition of the c-*MYC* rearrangement influenced the tumour's progression to a more aggressive subtype. However, we have no further histology reports in medical records to support this hypothesis. Also, we report a FCL (grade II) with involvement of t(8q24). The patient's previous biopsy showed a grade I FCL which recurred and progressed to a grade II FCL. The patient died 30 months later, which again reinforces the idea that the acquisition of this genetic anomaly may lead to the development of tumours with poor prognostic implications. Isolated case reports (Gauwerky et al, 1988; Lee et al, 1989) have appeared in the literature implicating c-*MYC* in the histological progression of some low-grade follicular lymphomas. Studies in transgenic mice provide an animal model for tumour progression in t(14;18) lymphoma, showing that evolution into a more aggressive lymphoma is accompanied by c-*MYC* rearrangements in half of the cases (McDonnell et al, 1991).

Recently, much interest has centred on a newly described gene, *BCL*-6, because of its involvement in a large subset of clinically important DLBC lymphomas. Our data showed rearrangement of *BCL*-6 to be the most frequent genetic abnormality detectable in our series of DLBC lymphomas (31%; 9 out of 29). This incidence is similar to previous reports – 29% of diffuse aggressive lymphomas reported by Otsuki et al (1995), 36% reported by LoCoco et al (1994) and 37% by Bastard et al (1994).

We detected 4% (1 out of 25) of FCLs to be positive for this rearrangement; Bastard et al (1994) reported this specific alteration in 13% (11 out of 84) and LoCoco et al (1994) in 6% (2 out of 31) of their respective series of follicular lymphoma cases.

The tumours with *BCL*-6 rearrangements were all of B-cell origin, and 50% had co-existing gene rearrangements. These involved the *BCL*-2 gene in three cases, the *BCL*-1 gene in one case and c-*MYC* in the remaining lymphoma. Two DLBC lymphomas had coexisting *BCL*-6 and *BCL*-2 rearrangements. This contrasts with the findings of LoCoco et al (1994) who report the absence of *BCL*-2 rearrangement in their series of *BCL*-6-rearrangement-positive DLBC (16 out of 45) lymphomas, hence suggesting that *BCL*-6 may define an independent pathway from *BCL*-2 in lymphomagenesis. However, our findings are substantiated by others, who showed coexisting *BCL*-2 and *BCL*-6 rearrangements in transformed lymphomas (17%; 9 out of 52) (Otsuki et al, 1995) as well as in de novo DLBC lymphomas (21%; 8 out of 39) (Bastard et al, 1994), thereby strongly suggesting that these two events are unlikely to be exclusive of one another.

We show coexisting *BCL*-2 and *BCL*-6 rearrangements in a DLBC lymphoma that had transformed from a low-grade FCL. The original biopsy specimen (FCL, grade I) also demonstrated a *BCL*-6 rearrangement. We also report a DLBC lymphoma demonstrating both *BCL*-1 and *BCL*-6 rearrangements that had an antecedent history of a low-grade lymphoma. The *BCL*-6 rearrangement status of the original biopsy is not known. The question of *BCL*-6 participating in the transformation process of some lymphoid malignancies remains unanswered; this needs to be resolved by accumulating and analysing more cases accompanied by clear medical history records. However, another consideration is the fact that the partner chromosomes of *BCL*-6 are numerous and may represent the sites of known, as well as of yet uncharacterized, gene loci perhaps harbouring unidentified genes, which may influence the progress of the process.

A DLBC lymphoma demonstrating both *BCL*-6 and c-*MYC* rearrangements was clinically aggressive, resulting in the patient's death 7 months after diagnosis. This might suggest that this combination of genetic events precipitates a tumour of poor prognostic implications.

In conclusion, our work has illustrated that associations exist between specific genomic alterations and defined subtypes of NHL. In addition, we report the interesting finding of coexisting rearrangements in a large percentage of the *BCL*-6-rearrangement-positive lymphoma cases. The identification of these molecular markers can be exploited in the diagnostic field, as well as aiding our understanding of the molecular pathogenesis of the diverse histological subtypes of NHL. Such markers may, in the future, be used to stratify lymphoma patients into different subgroups that share common genetic profiles. This novel classification scheme may then allow more accurate prediction of tumour biologies.

### ABBREVIATIONS

NHL, Non-Hodgkin's lymphoma; DLBC, diffuse large B cell lymphoma; FCL, follicle centre lymphoma; FL, follicular lymphoma; MCL, mantle cell lymphoma; SNCCL, small non-cleaved cell lymphoma; REAL, Revised European–American Lymphoma; WF, working formulation; FISH, fluorescence in situ hybridization; MTC, major translocation cluster

### ACKNOWLEDGEMENTS

The authors would like to thank Dr Amanda McCann and Dr Liz Moore for their advice in this paper. Also I am grateful to Dr Anna Migliazza and Dr Shinsake Iida for reviewing this manuscript. I would like to extend special thanks to Giorgio Cattoretti for his help in reviewing some histology slides. This work was in part supported by grants from the Irish Cancer Society as well as the Cancer Research Board.

### REFERENCES

Arnold A, Kim HG, Gaz RD, Eddy RL, Fukushima Y, Byers MG, Shows TB and Kronenberg HM (1989) Molecular cloning and chromosomal mapping of DNA rearranged with the parathyroid hormone gene in a parathyroid adenoma. *J Clin Invest* **83**: 2034–2040

Athan E, Foitl DR and Knowles DM (1991) BCL-1 rearrangement, frequency and clinical significance among B-cell chronic lymphocytic leukaemias and non-Hodgkin's lymphomas. *Am J Pathol* **138**: 591–599

© Cancer Research Campaign 1997

Bakhshi A, Jensen JP, Goldman P, Wright JJ, McBride DW, Epstein AL and Korsmeyer SJ (1985) Cloning the chromosomal breakpoint t(14;18) in human lymphomas: clustering around JH on chromosome 14 and near a transcriptional unit on 18. *Cell* **41**: 889–906

Baron BW, Nuciform G, McCabe N, Espinosa R, Lebeau MM and McKeithan T (1993) Identification of the gene associated with the recurring chromosomal translocation t(3;14)(q27;q32) and t(3;22)(q27;q11) in B-cell lymphomas. *Proc Natl Acad Sci USA* **90**: 5262–5266

Bastard C, Deweindt C, Kerckaret JP, Lenormand A, Rossi A, Pezzella F, Fruchart C, Duval C, Monconduit H and Tilly H (1994) LAZ3 rearrangement in non-Hodgkin's lymphoma. Correlation with histology, immunophenotype, karyotype, and clinical outcome in 217 patients. *Blood* **83**: 2423–2427

Berard CW and Dorfman RF (1974) Histopathology of malignant lymphomas. *Clin Haematol* **3**: 39–44

Clark HM, Jones DB and Wright DH (1992) Cytogenetic and molecular studies of t(14;18) and t(14;19) in nodal and extranodal B-cell lymphoma. *J Pathol* **166**: 129–137

Cleary ML and Sklar J (1985) Nucleotide sequence of a t(14;18) chromosomal breakpoint in follicular lymphoma and demonstration of a breakpoint cluster region near a transcriptionally active locus on chromosome 18. *Proc Natl Acad Sci USA* **90**: 5262–5266

Cotter FE (1990) The role of the BCL-2 gene in lymphoma. *Br J Haematol* **75**: 449–453

Croce CM and Nowell PC (1985) Molecular basis of human B-cell neoplasia. *Blood* **65**: 1–7

Dalla-Favera RM, Bregni M, Erickson J, Patterson D, Gallo RC and Croce CM (1982) Human c-*MYC* oncogene is located on the region of chromosome 8 that is translocated in Burkitt's lymphoma cells. *Proc Natl Acad Sci USA* **79**: 7824–7827

Deboer CJ, Loyson S, Kluin PM, Kluin-Nelemans HC, Schuuring E and Vankrieken JHJM (1993) Multiple breakpoints within the BCL1 locus in B-cell lymphoma: rearrangements of the cyclin D1 gene. *Cancer Res* **53**: 4148–4153

Dejong D, Voetdijk BHM, Van Ommen GLB, Kluin-Nelemans JC, Beverstock GC and Kluin PM (1989) Translocation t(14;18) in B-cell lymphomas as a cause for defective immunoglobulin production. *J Exp Med* **169**: 613–624

Donti E, Falina B, Pelicci PG, Donti GV, Rosetti A, Martelli M, Grignani F (1988) Immunological and molecular studies in a case of follicular lymphoma with an extra chromosome 12 and t(2;8) translocation. *Leukaemia* **2**: 41–44

Gauwerky CE, Haluska FG, Tsujimoto Y, Nowell PC and Croce CM (1988) Evolution of B-cell malignancy: pre B-cell leukaemia resulting from MYC activation in a B-cell neoplasm with a rearranged BCL-2 gene. *Proc Natl Acad Sci USA* **85**: 8548–8552

Gerard-Marchant R, Hamlin I, Lennert K, Rilke F, Stansfeld AG, Vanunnik JAM (1974) Classification of non-Hodgkin's lymphoma. *Lancet* **2**: 406–411

Graninger WB, Seto M, Boutain B, Goldman P and Korsmeyer SJ (1987) Expression of BCL-2 and BCL-2/Ig fusion transcripts in normal and neoplastic cells. *J Clin Invest* **80**: 1512–1516

Harris NL, Jaff ES, Stein H, Banks PM, Chan JKC, Cleary M, Delson G, Wolf-Peeters CD, Falini B, Gatter KC, Grogan TM, Isaacson PG, Knowles DM, Mason DY, Muller-Hermelink H, Pileri M, Ralfkiger E and Warnke RA (1994) A revised European–American classification of lymphoid neoplasms: a proposal from the international lymphoma study group. *Blood* **84**: 1361–1385

Hockenbery D, Nunez G, Milliman C, Schreiber RD and Korsmeyer SJ (1990) *BCL*-2 is an inner mitichondrial membrane that blocks programmed cell death. *Nature* **348**: 334–336

Hunter T and Pines J (1994) Cyclins and cancer. II. Cyclin D and CDK inhibitors come of age. *Cell* **79**: 573–578

Iida S, Rao P, Nallasivam P, Hibschoosh H, Butler M, Louie D, Dyomin V, Ohno H, Chaganti RSK and Dalla-Favera, R (1996) The t(9;14)(p13;q32) chromosomal translocation associated with lymphoplasmacytoid lymphoma involves the PAX-5 gene. *Blood* **88**: 4110–4117

Juneja S, Lukeis R, Cooper TI, Szelag G, Parkin JD, Ironside, Kerckaert JP, Deweindt C, Tilly H, Quief S, Lecocq G and Bastard C (1993) LAZ3, a novel zinc-finger encoding gene, is disrupted by recurring chromosome 3q27 translocations in human lymphomas. *Nature Genet* **5**: 66–70

Koduru PRK, Filippa DA and Richardson ME (1987) Cytogenetic and histologic correlations in malignant lymphoma. *Blood* **69**: 97–102

Komatsu H, Iida S, Yamamoto K, Mikuni C, Nitta M, Takahashi T, Ueda R and Seto M (1994) A variant chromosome translocation at 11q13 identifying PRAD1/cyclin D1 as the BCL-1 gene. *Blood* **84**: 1226–1231

Lambrechts AC, Deruiter PE, Dorssers LCJ and Van'tveer MB (1992) Detection of residual disease in translocation (14;18) positive non-Hodgkin's lymphoma, using the polymerase chain reaction: a comparison with conventional staging methods. *Leukaemia* **6**: 29–34

Lee JT, Innes DJ and Williams ME (1989) Sequential BCL-2 and c-MYC oncogene rearrangements associated with the clinical transformations of non-Hodgkin's lymphoma. *J Clin Invest* **84**: 1454–1459

Lenoir GM, Preud'homme JL, Bernheim A and Berger R (1982) Correlation between immunoglobulin light chain expression and variant translocation in Burkitt's lymphoma. *Nature* **298**: 474–476

Lococo F, Ye BH, Lista F, Corradini P, Offit K, Knowles DM, Chaganti RSK and Dalla-Favera R (1994) Rearrangements of the BCL6 gene in large cell non-Hodgkin's lymphoma. *Blood* **83**: 1757–1759

McDonnell TJ and Korsmeyer S (1991) Progression from hyperplasia to high grade malignant lymphoma in mice transgenic for the t(14;18). *Nature* **349**: 254–256

Medeiros LJ, Vankrieken JH, Jaffe ES and Raffeld M (1990) Association of *BCL*-1 rearrangements with lymphocytic lymphoma of intermediate differentiation. *Blood* **76**: 2086–2090

Miki T, Kawamata N, Arai A, Ohashi K and Aoki N (1994) Molecular cloning of the breakpoint for 3q27 translocation in B-cell lymphomas and leukaemias. *Blood* **83**: 217–222

Ohno H, Kerckaert JP, Bastard C and Fukuhara S (1994) Heterogeneity in B-cell neoplasms associated with rearrangement of the LAZ-3 gene on chromosome band 3q27. *Jpn J Cancer Res* **85**: 592–600

Osada H, Seto M, Ueda R, Emi N, Takagi N, Obata Y, Suchi T and Takahashi T (1989) BCL-2 gene rearrangement analysis in Japanese B cell lymphoma: novel BCL-2 recombination with immunoglobulin k chain gene. *Jpn J Cancer Res* **80**: 711–716

Otsuki T, Yano T, Clark HM, Bastard C, Kerckaert JP, Jaffe ES and Raffeld M (1995) Analysis of LAZ3(BCL-6) status in B-cell non-Hodgkin's lymphomas: results of rearrangement and gene expression studies and a mutational analysis of coding region sequences. *Blood* **85**: 2877–2884

Raffeld M and Jaffe S (1991) BCL-1, t(11;14), and mantle cell lymphomas. *Blood* **78**: 259–263

Sambrook J, Fritsch EF and Maniatis T (1989) *Molecular Cloning – A Laboratory Manual.* Cold Spring Harbor Laboratory Press:

Seto M, Jaeffer U, Hockett RD, Graininger W, Bennett S, Goldman P and Korsmeyer SJ (1988) Alternative promoters and exons, somatic mutation and deregulation of the BCL-2/Ig fusion gene in lymphoma. *EMBO* **7**: 123–131

Seto M, Yamamoto K, Iida S, Akao Y, Utsumi KR, Kubonishi I, Miyoshi I, Ohtsuki T, Yawata Y, Namba M, Motokura T, Arnold A, Takahashi T and Ueda R (1992) Gene rearrangement and overexpression of PRAD1 in lymphoid malignancy with t(11;14)(q13;q32) translocation. *Oncogene* **7**: 1401–1406

Stewart TA, Bellve AR and Leder P (1984) Transcription and promoter usage of the c-*MYC* gene in normal somatic and spermatogenic cells. *Science* **226**: 707–710

Taub R, Kirsch I, Morton C, Lenoir GM, Swan D, Tronick S, Aaronson S and Leder P (1982) Translocation of c-MYC gene into the immunoglobulin chain locus in human Burkitt's lymphoma and murine plasmacytoma cells. *Proc Natl Acad Sci USA* **79**: 7837–7841

Tsujimoto Y, Gorham J, Cossman J, Jaffe E and Croce CM (1985) The t(14;18) chromosomal translocation involved in B-cell neoplasms result from mistakes in VDJ joining. *Science* **229**: 1390–1393

Vaandrager JW, Schuuring E, Zwikstra E, Deboer CJ, Kleiverda KK, Vankrieken JM, Kluin-Nelemans C, Vanommen G, Raap AK and Kluin P (1996) Direct visualization of dispersed chromosomal translocations in mantle cell lymphoma by multicolour DNA fiber fluorescent in situ hybridization. *Blood* **88**: 1177–1182

Williams ME, Westermann CD and Swerdlow SH (1990) Genotypic characterization of centrocytic lymphoma: frequent rearrangement of the chromosome 11 *BCL*-1 locus lymphomas. *Blood* **76**: 1387–1391

Williams M, Meeker T and Swerdlow S (1991) Rearrangement of the chromosome 11 BCL-1 locus in centrocytic lymphoma. Analysis with breakpoint probes. *Blood* **78**: 493–498

Working Formulation (1982) Non-Hodgkin's lymphoma pathologic classification project. National Cancer Institute sponsored study of classification of non-Hodgkin's lymphoma: summary and description of a working formulation for clinical usage. *Cancer* **49**: 2112

Wotherspoon AC, Pan L, Diss TC and Isaacson PG (1990) A genotypic study of low grade B-cell lymphomas, including lymphomas of mucosa associated lymphoid tissue (MALT). *J Pathol* **162**: 135–140

Yano T, Jaffe E, Longo DL and Raffeld M (1992) MYC rearrangements in histologically progressed follicular lymphomas. *Blood* **80**: 758–767

Ye BH, Rao PH, Chaganti RSK and Dalla-Favera R (1993*a*) Cloning of BCL-6, the locus involved in chromosome translocations affecting band 3q27 in B-cell lymphomas. *Cancer Res* **53**: 2732–2735

Ye BH, Lista F, Lococo F, Knowles DM, Offit K, Chaganti RSK and Dalla-Favera R (1993*b*) Alteration of a zinc finger encoding gene, BCL-6 in diffuse large cell lymphoma. *Science* **262**: 747–750

© Cancer Research Campaign 1997